# Partially coherent ultrafast spectrography

**DOI:** 10.1038/ncomms7465

**Published:** 2015-03-06

**Authors:** C. Bourassin-Bouchet, M.-E. Couprie

**Affiliations:** 1 Synchrotron SOLEIL, Saint Aubin, BP 34, 91 192 Gif-sur-Yvette, France; 2 Laboratoire Charles Fabry, UMR 8501, Institut d’Optique, CNRS, Univ Paris Sud 11, 2, Avenue Augustin Fresnel, 91127 Palaiseau Cedex, France

## Abstract

Modern ultrafast metrology relies on the postulate that the pulse to be measured is fully coherent, that is, that it can be completely described by its spectrum and spectral phase. However, synthesizing fully coherent pulses is not always possible in practice, especially in the domain of emerging ultrashort X-ray sources where temporal metrology is strongly needed. Here we demonstrate how frequency-resolved optical gating (FROG), the first and one of the most widespread techniques for pulse characterization, can be adapted to measure partially coherent pulses even down to the attosecond timescale. No modification of experimental apparatuses is required; only the processing of the measurement changes. To do so, we take our inspiration from other branches of physics where partial coherence is routinely dealt with, such as quantum optics and coherent diffractive imaging. This will have important and immediate applications, such as enabling the measurement of X-ray free-electron laser pulses despite timing jitter.

## Abstract (web summary)

Ultrafast metrology typically relies on pulse coherence, but full coherence is not always possible in emerging attosecond and ultrashort X-ray technologies. Here, Bourassin-Bouchet and Couprie adapt frequency-resolved optical gating (FROG) to measure partially coherent optical pulses in the attosecond scale.

## Graphical abstract

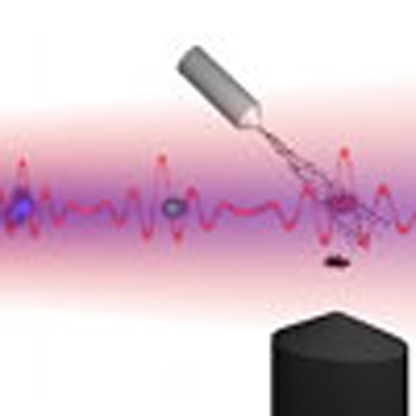

Coherence is one of the most fundamental concepts in physics and is intimately related to the notion of measurement. Partial coherence arises when the observed quantity, for example, a light field, varies during the measurement process in degrees of freedom that cannot be resolved by the detection device. It thus determines if a clear signal, such as an interference pattern, can emerge from the measurement. In ultrafast metrology, this imposes to keep the waveform under test as stable as possible (in space, in polarization, over successive shots) during the measurement^2^. Such a stringency is sometimes impossible to fulfil, especially for emerging ultrashort extreme-ultraviolet and X-ray (XUV) sources, such as sources based on high-harmonic generation (HHG)^3^,^4^,^5^ or free-electron lasers (FEL)^6^,^7^. However, some other domains of physics have solved the problem of performing a measurement in the presence of partial coherence. In quantum optics (QO), decoherence drastically limits the lifetime of qubits by changing any pure quantum state into a statistical state mixture. Therefore, techniques allowing one to monitor^8^ and control^9^ the coherence of quantum systems have become an area of tremendous interests. In coherent diffractive imaging (CDI), a lensless microscopy technique allowing the reconstruction of nanometric samples from diffraction measurements, strong efforts have been made to maintain the quality of the imaging in the presence of partially coherent light^10^,^11^,^12^.

In this article, we show that solutions developed for QO and CDI can be adapted to frequency-resolved optical gating (FROG), the well-known spectrographic pulse measurement technique^13^, thus enabling the temporal metrology of partially coherent pulses. The elegance of this approach lies in the fact that no experimental modification is required; only the processing of the measurement changes. Although applicable to virtually any FROG measurement, we focus on applications for the metrology of ultrashort XUV pulses with FROG for the complete reconstruction of attosecond bursts (FROG-CRAB)^14^. First, we explain the principle of this technique, named Mixed-FROG. We then show that conventional pulse measurement techniques can be strongly misled when partial coherence arises, and how Mixed-FROG solves this problem. Finally, we give two examples of applications for probing the space–time structure of attosecond pulses, or measuring FEL pulses in the presence of temporal jitter.

## Results

### Adapting FROG to partial coherence

**Figure 1:**
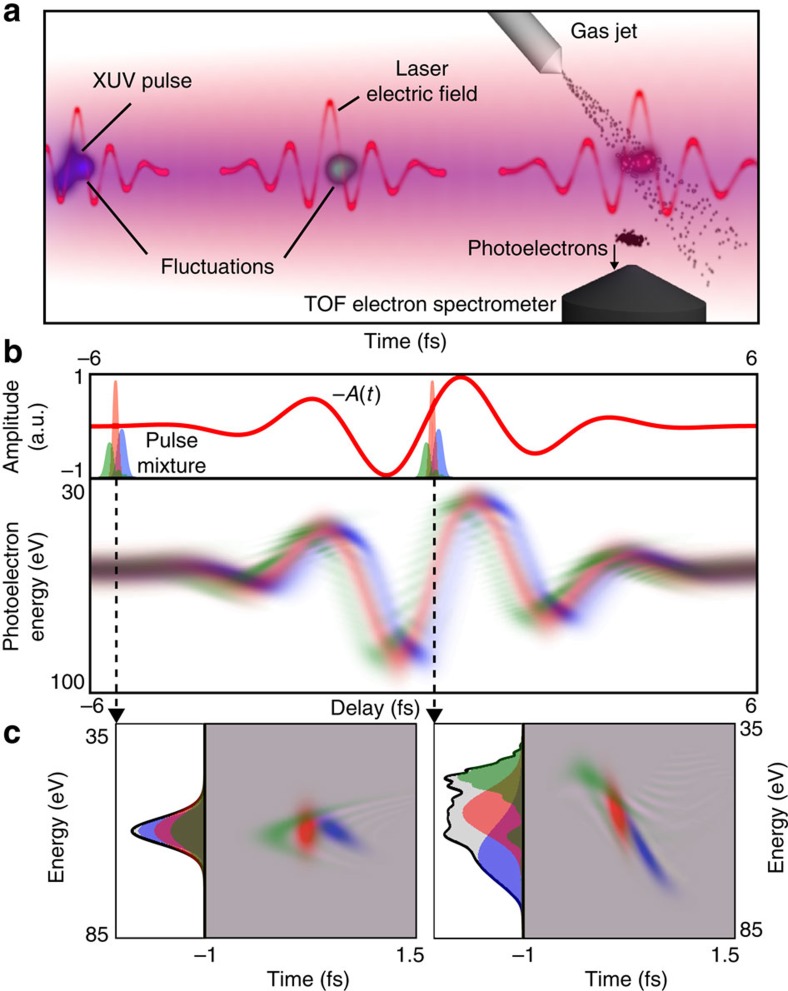
Partially coherent spectrography with ultrashort XUV pulses. (**a**) Schematic of a FROG-CRAB measurement in the presence of decoherence, here due to shot-to-shot XUV pulse fluctuations. (**b**) Photoelectron spectra obtained for various delays between the pulse mixture and the 800-nm laser pulse (duration 3-fs FWHM, intensity 8 TW cm^−2^) represented by its vector potential *A*(*t*). Each colour (red, green and blue) corresponds to a pure ultrashort pulse. (**c**) Wigner function *W*_P_ (*ω*, *t*) and the associated energy spectrum of the electron wave packet without (left panel) and with (right panel) laser pulse at a given delay.

In regular ‘pure state’ FROG spectrography, one measures the spectrally resolved cross-correlation of an ultrashort pulse, represented by the complex field *P*(*t*), with a second pulse *G*(*t*−*τ*) referred to as the gate. The resulting spectrogram 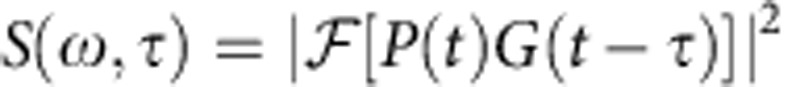, where 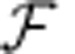 denotes the Fourier transform with respect to *t*, is then numerically processed in order to extract both *P*(*t*) and *G*(*t*). In the specific case of FROG-CRAB, an XUV pulse focused into a gas jet frees an electron wave packet (EWP) that replicates the pulse properties. An infrared laser pulse synchronized with the XUV pulse then modulates the EWP, the spectrum of which is measured with a time of flight (TOF) spectrometer while scanning the infrared/XUV delay, see Fig. 1a. The result of this measurement takes the form of a spectrogram, where the EWP plays the role of the pulse *P*(*t*) and where the gate *G*(*t*) corresponds to a phase modulation induced on the EWP by the laser field.

In practice, the EWP can accumulate decoherence originating from the XUV pulse (due to, for example, fluctuations in space or from shot to shot), from photoionization (for example, electron–ion collisions) or from the detection device (the spectrometer response). The final spectrogram ‹*S*› is then averaged over all the wave packets that participated to the measurement:


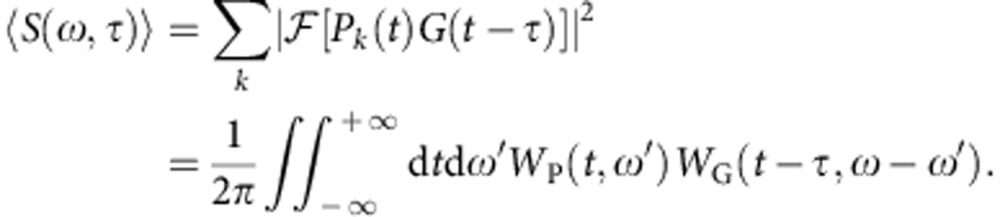


In that case, the conventional spectrogram inversion procedure fails since the ultrashort pulse no longer corresponds to a well-defined electric field *P*(*t*). One must instead describe it with the help of statistical quantities, such as the two-time correlation function *C*(*t*, *t*′)=‹*P*(*t*)P*(*t*′)› (usually referred to as the density matrix in QO) or the Wigner representation *W*_P_ in the chronocyclic space (*ω*, *t*).

Adapting FROG to partial coherence requires extracting these quantities from the spectrogram. This can be understood through two equivalent reasonings. First, the average spectrogram can be seen as the incoherent superposition of characteristic pure spectrograms 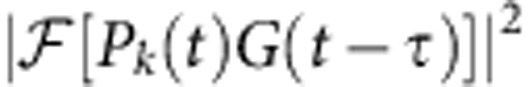, distinguished by their colour in Fig. 1b. Retrieving 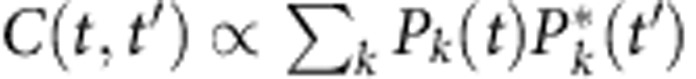 therefore requires the extraction of a few coherent pulses *P*_*k*_(*t*), which is made possible by the large amount of redundant information in ‹*S*›. Second, the problem can be understood in terms of chronocyclic tomography^15^, the equivalent in ultrafast optics of quantum state tomography in QO^8^. Each spectrum composing the spectrogram corresponds to a projection of *W*_P_ (*ω*, *t*) along the *ω* axis (see Fig. 1c). The gate, represented by its Wigner function *W*_G_, deforms *W*_P_ and its projection in a different way for each delay. It is then possible to reconstruct *W*_P_ tomographically from these spectra.

For this process to succeed, the spectrogram needs to be a quorum, which, in quantum state tomography, represents a finite set of measurements that suffices to retrieve the state^16^. Indeed, the key is to measure projections of *W*_P_ that are diversified enough to describe the complexity of the mixture. Consider the example in Fig. 1b,c, where a mixture of attosecond pulses is confined within half an infrared optical cycle around a zero of the laser vector potential **A**, that is the so-called streaking regime^17^. If the electrons are collected in the direction of polarization of the laser field, the state *W*_P_(*t*, *ω*±*p*_0_*A*_0_*t*) is streaked, *p*_0_ being the momentum of the unstreaked electrons and *A*_0_ the amplitude of **A**, and the measured spectrum reveals details about the mixture that were hidden in the absence of gate. If *A*_0_ is large enough, one operates a time-to-frequency conversion (TFC). The spectrum ‹*S*(*ω*)›≈‹|*P*(±*p*_0_*A*_0_*t*)|^2^› directly maps the time profile of the mixture^18^, and thus corresponds, up to a scaling factor, to the temporal marginal of *W*_P_ (ref. 1). A FROG-CRAB trace containing all the spectra from the unstreaked case to the TFC will fully cover the chronocyclic space associated with the EWP. Leaving aside experimental considerations about noise and data-sampling rate, this condition ensures the trustworthy reconstruction of an arbitrary pulse mixture^15^. As will be shown below, it is not mandatory to reach the TFC, nor to lie in the streaking regime, to obtain an accurate reconstruction if *W*_P_ can be properly described by a smaller set of projections. In practice, a good strategy will be to maximize *A*_0_, that is the laser intensity, to obtain projections as diversified as possible.

As a last step, one must identify the most robust way to extract the mixture from the data in practice, ideally by adapting the Principal Component Generalized Projections Algorithm (PCGPA) widely used to inverse FROG traces^19^. To do so, we draw our inspiration from ptychography^20^. In this form of CDI, the sample is numerically retrieved from diffraction patterns obtained for different positions of the sample across the focal spot of an X-ray beam. The methodology appears to be the same as in FROG^21^: performing intensity measurements in the far field/frequency domain while changing the overlap between the two distributions in the near field/time domain. In CDI, a key requirement is to illuminate the object with a highly coherent beam, and the quality of the imaging rapidly collapses when the coherence of the probe beam decreases. However, a recent breakthrough has shown that ptychographic sample reconstructions were still achievable with a partially coherent X-ray beam if processing the diffraction patterns with adapted algorithms^11^. Especially, the difference map algorithm turns out to rely on the same characteristic steps as the PCGPA. We take advantage of this analogy to develop a new FROG algorithm, which we refer to as the Generalized Projection Algorithm for Mixed States or MSGPA. In practice, the algorithm reconstructs several pure spectrograms in parallel in order to retrieve the few characteristic pulses *P*_*k*_(*t*) associated with the mixture along with the gate *G*(*t*). The detailed principle is reported in Supplementary Note 1 and Supplementary Fig. 1. This general approach is not restricted to the PCGPA, and should also enable one to adapt other FROG algorithms to partial coherence^22^. For simplicity, we hereafter refer to this extension of FROG to mixed states as Mixed-FROG.

### The coherent artifact in attosecond metrology

Decoherence is often present in attosecond metrology^23^. As the approaches used so far are relevant only if the pulse is fully coherent, they lead to erroneous reconstructions when partial coherence arises. This problem is known as the coherent artifact in ultrashort laser metrology^24^,^25^. Consider for example the RABBIT technique (Reconstruction of Attosecond Beating By Interference of two-photon Transitions) used for characterizing attosecond pulse trains obtained via HHG^3^,^26^. The EWP is dressed with a low-intensity laser pulse, which induces two-photon XUV-infrared transitions. This leads to the creation of sidebands that interfere and encode the phase difference between the two neighbouring harmonics. As with any interference pattern, the visibility *V* of a sideband gives direct insight into the degree of coherence of the interfering fields. In the fully coherent case, 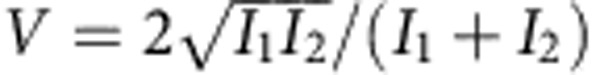, with *I*_1,2_ the intensities of the two consecutive harmonics, and is generally ≃0.9. Experimentally, such a high visibility is rarely obtained^3^, which tends to show that partial coherence is usually present in practice.

**Figure 2:**
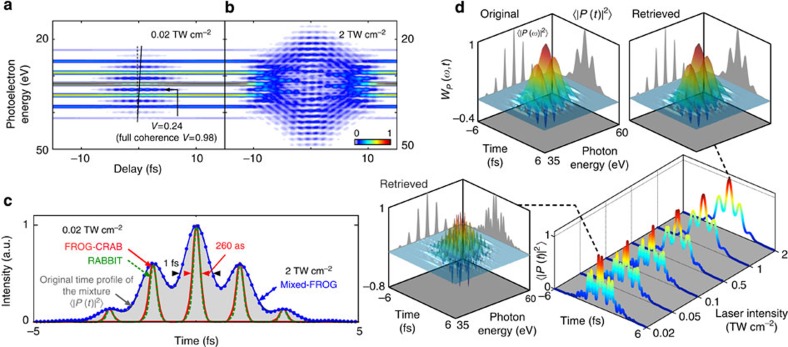
Low coherence RABBIT measurement. RABBIT trace in the presence of partial coherence for a laser intensity of (**a**) 0.02 TW cm^−2^ and of (**b**) 2 TW cm^−2^. The tilted black line in **a** indicates the variation of the attosecond group delay, the effective and coherent contrasts *V* of one of the sidebands are also reported. (**c**) Attosecond pulse train retrieved from the trace in **a** using FROG-CRAB (red continuous line) and RABBIT (green dashed line), and retrieved by Mixed-FROG (blue circles) from the trace in **b**. The grey shaded curve represents the temporal marginal of the original Wigner function *W*_P_ (*ω*, *t*) of the mixture. (**d**) Temporal marginals retrieved with Mixed-FROG for different laser intensities. Inset: original and retrieved Wigner functions of the attosecond pulse train.

To reproduce this observation, we simulate a low coherence RABBIT trace in Fig. 2a for an infrared intensity of 0.02 TW cm^−2^. In this example, decoherence is chosen to arise from fluctuations of the XUV pulse (see Supplementary Fig. 2 and Supplementary Methods). The pulse extraction can be carried out either by using the RABBIT procedure, the black line in Fig. 2a giving the attosecond group delay dispersion, or through FROG-CRAB by using the PCGPA^27^. Both RABBIT and FROG-CRAB retrieve a train composed of ~260-as full-width at half-maximum (FWHM) pulses that are temporally well separated. However, the actual time profile of the mixture ‹|*P*(*t*)|^2^› appears to be strongly affected by decoherence, the main spike having a duration of ~1-fs FWHM. As in near-visible laser metrology, the coherent artifact can strongly bias attosecond pulse measurements.

We now use Mixed-FROG to retrieve the pulse mixture associated with the RABBIT trace. The Wigner function depicted in Fig. 2d) for a laser intensity of 0.02 TW cm^−2^ is obtained. The reconstruction is not satisfactory as the obtained Wigner function does not match the original one, see Supplementary Fig. 3. This means that the RABBIT trace is not a quorum, that is, that the projections of *W*_P_ generated by the gate are not varied enough to reveal the finest details of the Wigner function. Indeed, only consecutive harmonics are interfered in RABBIT, whereas getting the full coherence information requires interfering each harmonic with all the others. By increasing the laser intensity, one creates extra sidebands that correspond to the absorption or emission of several infrared photons after the absorption of the XUV photon. These novel sidebands interfere, which encodes in the trace the correlation information between nonconsecutive harmonics. The temporal marginals retrieved for various laser intensities are shown in Fig. 2d. Beyond 0.5 TW cm^−2^, the retrieved profile becomes independent of the laser intensity, indicating that the trace contains sufficient information about the mixture. This is confirmed by the agreement between the original and retrieved Wigner functions. By its unique ability to dissociate coherent and incoherent contributions in the spectrogram, Mixed-FROG retrieves the actual profile and duration of pulse mixtures, and solves the problem of the coherent artifact in ultrafast optics.

### Probing the attosecond space–time structure

**Figure 3:**
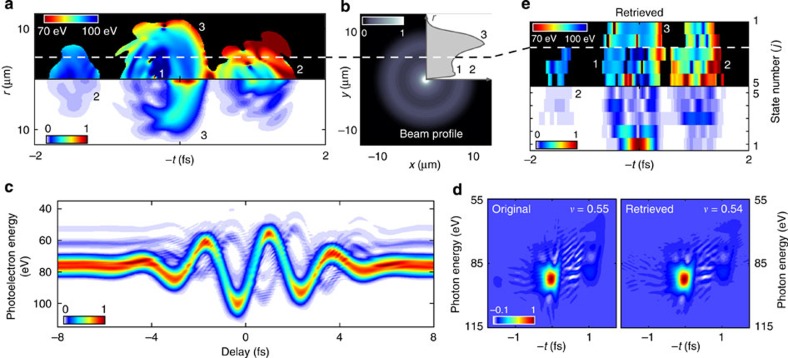
Space–time distortions of an isolated attosecond pulse. (**a**) Spatiotemporal electric field of an isolated attosecond pulse. The instantaneous frequency and the field modulus are depicted. (**b**) Corresponding two-dimensional (2D) spatial profile of the beam (inset: spatial profile integrated angularly). (**c**) Spectrogram obtained from the pulse in **a**. (**d**) Original and retrieved Wigner functions of the attosecond pulse. (**e**) Instantaneous frequency and modulus of the five most intense coherent states in the mixture.

From the moment it can be diagnosed, partial coherence is no longer a detrimental phenomenon in ultrafast optics, and can instead be used to probe fundamental processes. If it has not been modified by parasitic effects from the beam line^28^, the space–time structure of an attosecond pulse is a unique signature of the physical phenomena occurring during its birth^29^, as exemplified in Fig. 3a. The isolated attosecond pulse *P*(*t*, *r*) is generated by HHG by focusing the driving pulse after a neon gas jet to emphasize contributions of long quantum trajectories^30^. As highlighted by its instantaneous frequency *ω*(*t*, *r*)=−∂*ϕ*/∂*t*, with *ϕ*(*t*, *r*)=Arg(*P*), the field can be summarized into two parts delimited by the white dashed line in Fig. 3a,b. Near the axis, both short and long trajectories contribute to the emission. The resulting pulse profile consists of a main ~95-eV pulse (dark blue) with a complex temporal structure (1) surrounded by a red pre-pulse and a weak post-pulse (2). Off axis, where only long trajectories contribute, the central pulse subsists and its temporal shape becomes more regular with a central photon energy near 90-eV (3). Even though the maximum XUV peak intensity is reached on-axis (1–2), most of the energy is in fact contained in an outer ring, see Fig. 3b, that is, in the off-axis part of the central pulse (3).

The photoionization process occurring in every point of the XUV beam, the EWP reproduces the local structure of the pulse. However, the TOF spectrometer does not have a spatial resolution and integrates incoherently photoelectrons coming from different points in space. Therefore, an incoherent averaging occurs over the transverse spatial dimensions. Considering that the infrared-dressing beam is spatially homogenous with an intensity of 5 TW cm^−2^, one obtains the trace in Fig. 3c. This spectrogram and the associated Wigner function in Fig. 3d contain complex coherent and incoherent features. The dark blue core of the central pulse (1) arrives at the same transverse position as the pre- and post-pulses (2) in the gas jet. Consequently, it interacts with the same group of atoms during photoionization, and is thus coherent with the two satellite pulses, as highlighted by the fringes visible in Fig. 3c,d. However, the wings of the central pulse (3) exhibit a different temporal profile and do not contain satellite pulses. They thus generate a different spectrogram that is incoherently superposed with the one coming from the centre of the beam. As shown in Fig. 3d, the Wigner function retrieved with Mixed-FROG agrees very well with the original one, see Supplementary Movie 1.

Equivalently to the spectral decomposition of the density matrix in QO, one can conveniently expand *C*(*t*, *t*′) into its eigenstates, and estimate the purity 
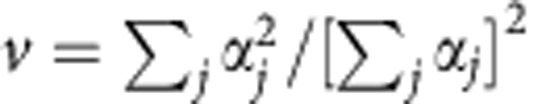
 of the mixture, where *α*_*j*_ denotes the associated eigenvalues. The eigenstates of the retrieved mixture, sorted in descending *α*_*j*_, are depicted in Fig. 3e. One can notice two distinct behaviours: the pre- and post-pulses (2) are contained in the same coherent states (*j*=3, 4, 5) as the blue core of the central pulse (1), indicating that they originate from the same transverse position. States *j*=1, 2, however, correspond to well-confined pulses with negligible satellite pulses. As they represent the two states carrying most of the beam energy (the largest eigenvalues), they are to be located in the intense outer ring (3). Therefore, the modal expansion gives insight into the attosecond spatiotemporal structure, as different coherent states correspond to different transverse positions in the beam. On a more general basis, this shows how partial coherence can be used as a powerful information vector that becomes accessible with Mixed-FROG.

### Temporal measurement of FEL pulses

The temporal metrology of femtosecond XUV pulses from FELs has become an area of tremendous interest^18^,^31^. However, the poor shot-to-shot repeatability of these pulses has prevented so far their full amplitude and phase characterization, and has made impossible the transposition of FROG-CRAB to FEL. Indeed, for a FROG-CRAB measurement to succeed, one requirement learned from attosecond metrology is that the infrared-dressing pulse must be synchronized with the XUV pulse with an accuracy much better than the infrared optical cycle (~2.6-fs)^5^. Even though seeded FELs can deliver pulses with a shape stable from shot-to-shot^7^, their synchronization with an external infrared laser remains challenging, so that in practice a temporal jitter of 5- to 100-fs (refs 31, 32) naturally exists between the two beams, preventing the pulse measurement. Once again, the fundamental problem comes from the fact that conventional approaches are unable to manage the partial longitudinal coherence of FEL pulses.

**Figure 4:**
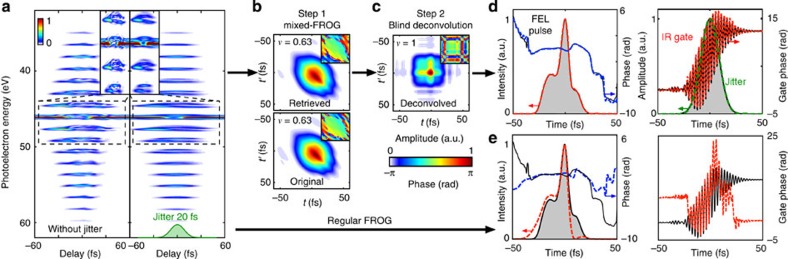
Free-electron laser pulse measurement in the presence of temporal jitter (**a**) Spectrogram of an FEL pulse dressed by a 20-fs laser pulse without and with temporal jitter between the infrared and XUV pulses. The green shaded area indicates the jitter envelope. Insets represent a zoom in of the area delimited by the dashed line. A two-step procedure is used to retrieve the FEL pulse from the spectrogram in **a** in the presence of jitter. (**b**) First, Mixed-FROG is used to retrieve the FEL pulse correlation function *C*(*t*, *t*′). (**c**) Second, a blind deconvolution is performed to suppress the influence of jitter on *C*(*t*, *t*′). (**d**) Original (black continuous curve and grey shaded area) and retrieved (coloured dashed lines) XUV pulse, jitter envelope and infrared phase modulator. (**e**) XUV pulse and infrared gate retrieved through the conventional FROG-CRAB technique.

Mixed-FROG enables the full characterization of seeded FEL pulses from jitter-averaged spectrograms. We illustrate such a scenario in Fig. 4. The XUV pulse has been calculated to replicate the conditions of the LUNEX5-seeded FEL project^34^,^35^. The infrared and XUV spectra being both narrower than the infrared photon energy (~1.5-eV), the spectrogram exhibits characteristic sidebands that are well-isolated spectrally^36^,^37^, as shown in Fig. 4a. When accounting for the inherent optical/XUV temporal jitter (20-fs FWHM gaussian envelope), fine details in the sidebands disappear.

Using Mixed-FROG, the correlation function *C*(*t*, *t*′) in Fig. 4b is retrieved. The convergence of the algorithm is depicted in Supplementary Fig. 4. However, the influence of jitter prevents a direct access to the underlying FEL pulse. In QO experiments, when the main sources of decoherence are known, one can model and subtract their influence on the mixture in order to retrieve the prepared quantum state^38^. We follow the same strategy here, and consider that jitter is the sole source of decoherence, that is, that the mixture is composed of identical waveforms *P*(*t*) (the FEL pulse to retrieve) randomly delayed in the jitter envelope *J*(*t*), see Supplementary Fig. 5. Despite its simplicity, this assumption sufficiently constraints the shape of *C*(*t*, *t*′) to enable the unambiguous retrieval of *P*(*t*) and *J*(*t*). In the end, the whole process essentially consists of operating a blind deconvolution of the retrieved correlation function. Using the algorithm described in Supplementary Note 2 and Supplementary Fig. 6, we disentangle the influence of the temporal jitter and of the pulse, see Fig. 4c. The results of this two-step ‘Mixed-FROG + deconvolution’ process are summarized in Fig. 4d. The FEL pulse, the jitter envelope and the infrared gate agree very well with the original fields. As a comparison, the pulse and gate obtained with the regular FROG-CRAB procedure are depicted in Fig. 4e.

These results demonstrate that the FEL pulse retrieval is still possible, even though the jitter is one order of magnitude larger than the infrared optical cycle, which will dramatically relax the timing requirements in FEL pulse metrology. In the case of FEL relying on Self-Amplified Spontaneous Emission^39^, the deconvolution step will no longer be adapted as the pulse shape changes on a shot-to-shot basis. Instead, Mixed-FROG will provide the full statistics of XUV waveforms (amplitude and phase) that were accumulated during the trace acquisition. Moreover, the approach consisting in accounting for known sources of decoherence to retrieve the underlying pulse applies to many situations. For instance, the resolution of the TOF spectrometer is another common source of decoherence^27^,^37^. As shown in Supplementary Note 2 and Supplementary Fig. 7, a simple modelling of the mixture can enable the joint measurement of the spectrometer resolution along with the pulse and gate.

## Discussion

By combining numerical tools from CDI with the formalism used in QO, we reconstruct partially coherent ultrashort pulses from conventional FROG spectrograms. The proposed solution goes far beyond current state-of-the-art metrology and gives access to a much richer physics. As Mixed-FROG does not require any change on the data-acquisition side, it offers a manifold of applications immediately accessible. In attosecond physics, it will allow one to determine up to which extent the coherence of the optical wave packet is transferred to the EWP during photoionization^40^,^41^. Moreover, the role of the pulse and gate being symmetric in the spectrogram, the presented approach could be reversed to probe mixtures of infrared electric fields with XUV pulses. However, Mixed-FROG carries even greater promises for the metrology of near-visible pulses, especially in applications requiring highly coherent ultrashort pulses, such as coherent quantum control in molecules^42^, the coherent addition of fibre amplifiers^43^,^44^, or in optical telecommunications^45^. It will also enable the study of decoherence during complex nonlinear processes, such as supercontinuum generation^46^ and optical rogue waves^47^. Partially coherent spectrography redefines the notion of pulse measurement in ultrafast optics, and will potentially have an impact on other domains where spectrograms are routinely used, including acoustics and seismography^48^,^49^.

## Methods

### Simulation of the isolated attosecond pulse

The simulation is performed with a rotational symmetry. The driving pulse is an 800-nm Fourier-limited gaussian pulse with a duration of 4 fs (intensity FWHM). The pulse with a gaussian spatial profile and a radius of 2.5 cm (1/*e*^2^) is focused (*f*=2 m) 0.5 mm after a neon gas jet. The pulse peak intensity at focus equals 5 × 10^14 ^W cm^−2^. The gas jet has a supergaussian density profile (0.5 mm FWHM) and a pressure of 10 mbar. The interaction is simulated with a nonadiabatic code^50^, and the single-atom response is calculated within the strong-field approximation. The radiation outgoing the jet is then filtered with a 400-nm zirconium foil and reflected off a molybdenum–silicon multilayer mirror (layer thicknesses: Mo 3.5 nm, Si 3.5 nm, 8 periods, 5° incidence angle) in order to select the 80- to 100-eV bandpass near the cutoff region. Perfect imaging conditions are assumed, so that the XUV pulse focused in the TOF spectrometer exhibits the same space–time distortions as the pulse outgoing the generation medium.

### Free-electron laser pulse simulation

Using the GENESIS code^51^, the performances of the LUNEX5 FEL are simulated in the Echo-Enabled Harmonic Generation scheme^52^ at 20 nm extensively described in ref. 34. The seed pulse (30 fs FWHM, 266 nm central wavelength) is divided into two beams injected in the first (respectively, second) modulator with a peak power of 15 MW (respectively, 30 MW). The energy of the electron beam is set to 400 MeV, with the first two radiator sections on. The outgoing XUV pulse, centred at 20 nm, has a duration of 10-fs FWHM and a peak power of 70 MW.

## Author contributions

C.B.-B. developed the concept of partially coherent spectrography and the algorithms, performed the numerical simulations and wrote up the manuscript. M.-E.C. supervised the project.

## Additional information

**How to cite this article:** Bourassin-Bouchet, C. & Couprie, M.-E. Partially coherent ultrafast spectrography. *Nat. Commun.* 6:6465 doi: 10.1038/ncomms7465 (2015).

## Supplementary Material

Supplementary Information Supplementary Figures 1-7, Supplementary Notes 1-2, Supplementary Methods and Supplementary References

Supplementary Movie 1 Convergence of the MSGPA on the mixture used in Fig. 3 in the main text. Left panels: original and retrieved spectrograms. Upper central panel: temporal marginal of the original mixture (grey shaded curve) and square modulus of the retrieved pulses. Lower central panel: original (black curve) and retrieved (red curve) phase of the gate. Right panels: original and retrieved Wigner distributions of the XUV pulse mixture.
