# Supplementary material for: Partially coherent ultrafast spectrography
Source: Nat Commun. 2015 Mar 6;6:6465. doi: 10.1038/ncomms7465 (PMC4366527; doi:10.1038/ncomms7465)
Supplement: Supplementary Information — Supplementary Figures 1-7, Supplementary Notes 1-2, Supplementary Methods and Supplementary References [file ncomms7465-s1.pdf]

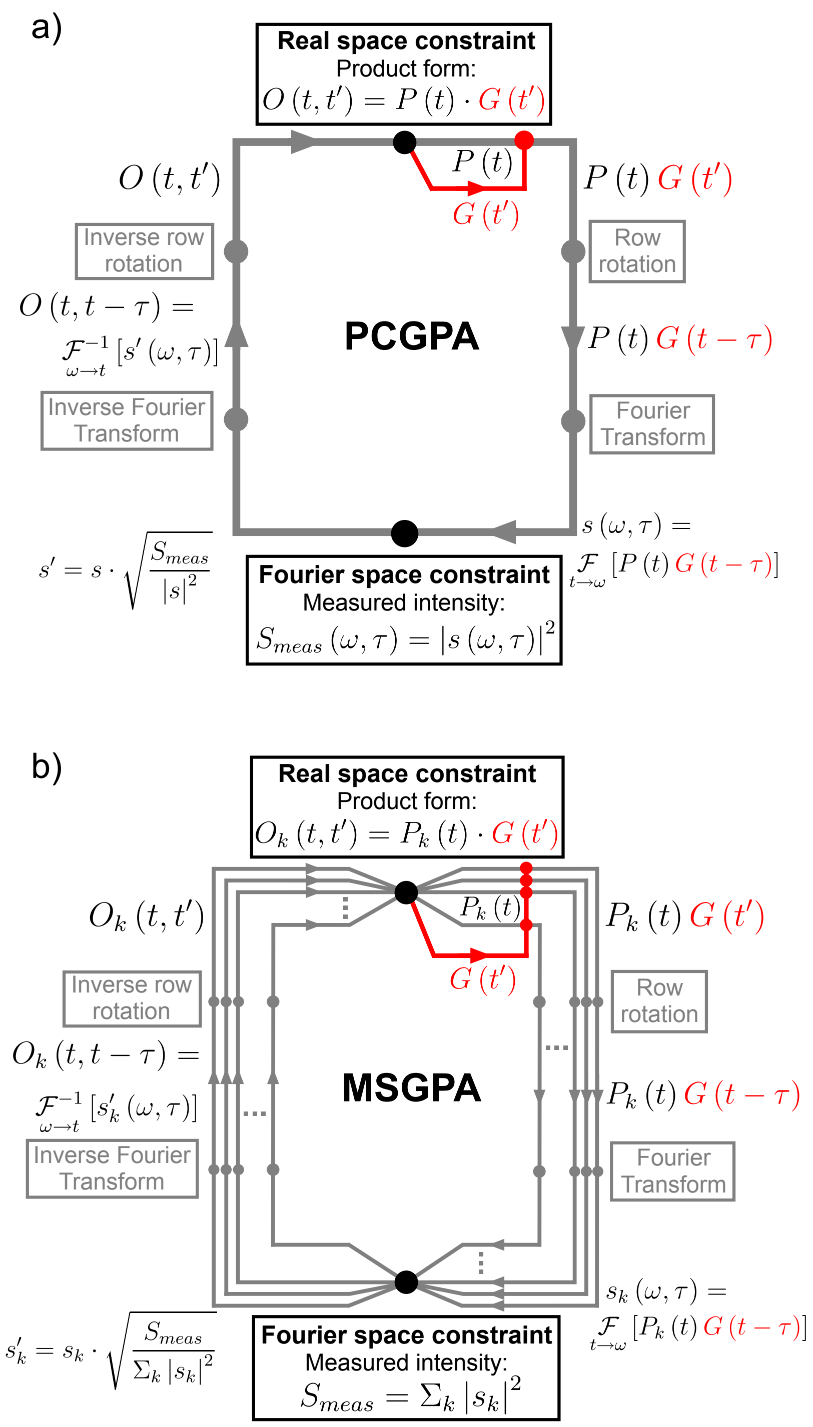

Supplementary Figure 1: **Comparison between the PCGPA and MSGPA** The main algorithmic steps are summarised in a) for the Principal Components Generalised Projections Algorithm (PCGPA), and in b) for the Mixed-States Generalised Projections Algorithm (MSGPA).

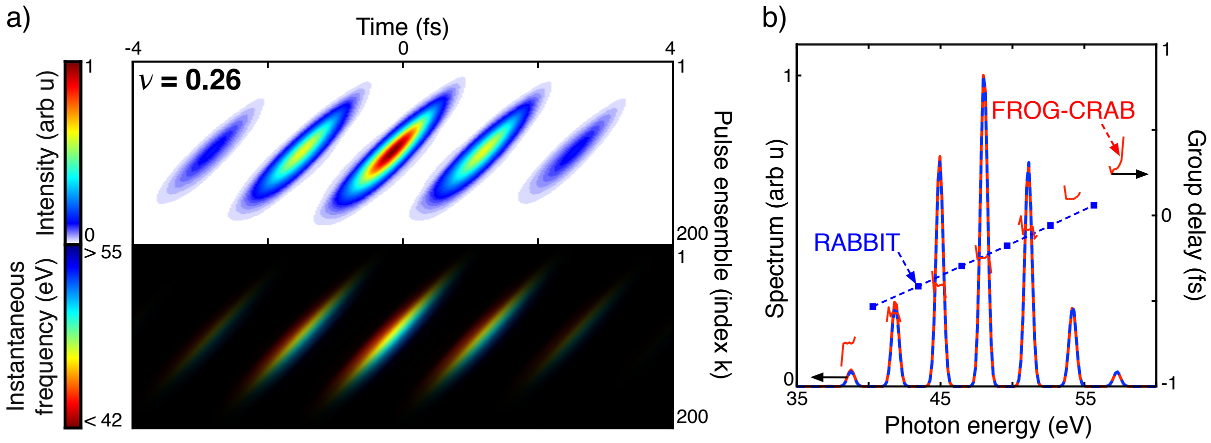

Supplementary Figure 2: **Mixture used for the simulation of the RABBIT trace** a) Ensemble of attosecond pulse trains composing the mixture used to simulate the RABBIT trace (upper panel : pulse intensity, lower panel : instantaneous frequency). b) Spectrum and group delay retrieved with RABBIT (blue dashed lines) and FROG-CRAB (red continuous lines) from the trace obtained for a dressing intensity of $0.02\,\mathrm{TW\,cm^{-2}}$ shown in Fig. 2 a) in the main text.

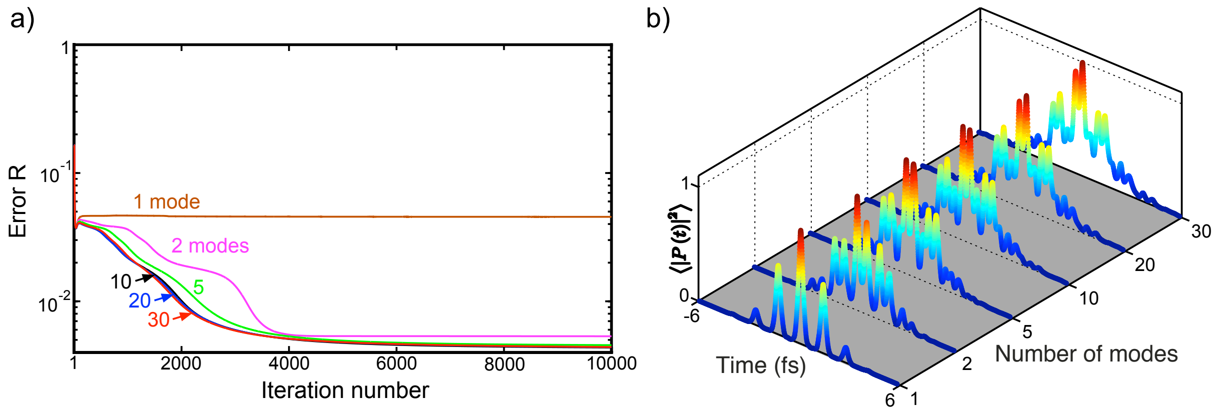

Supplementary Figure 3: **Effect of the number of modes on the reconstruction** a) Evolution of the error R between the original and retrieved spectrograms when the RABBIT trace (Fig. 2 a) in the main text, dressing intensity $0.02\,\mathrm{TW\,cm^{-2}}$) is processed with the MSGPA using 1, 2, 5, 10, 20 and 30 modes for the reconstruction. In b) are shown the temporal marginals retrieved in each case.

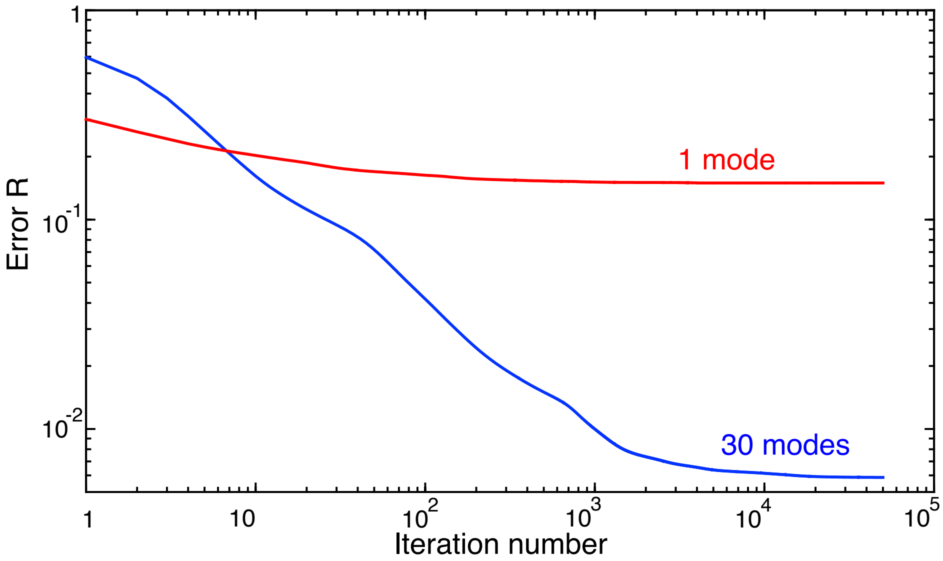

Supplementary Figure 4: Processing of the jitter-averaged spectrogram of the FEL pulse with the MSGPA and PCGPA The trace described in the right panel of Fig. 4 a) in the main text is processed. The error R between the original $\langle S \rangle_O$ and retrieved $\langle S \rangle_R$ spectrograms is plotted as a function of the iteration number with 1 mode (PCGPA) and with 30 modes (MSGPA).

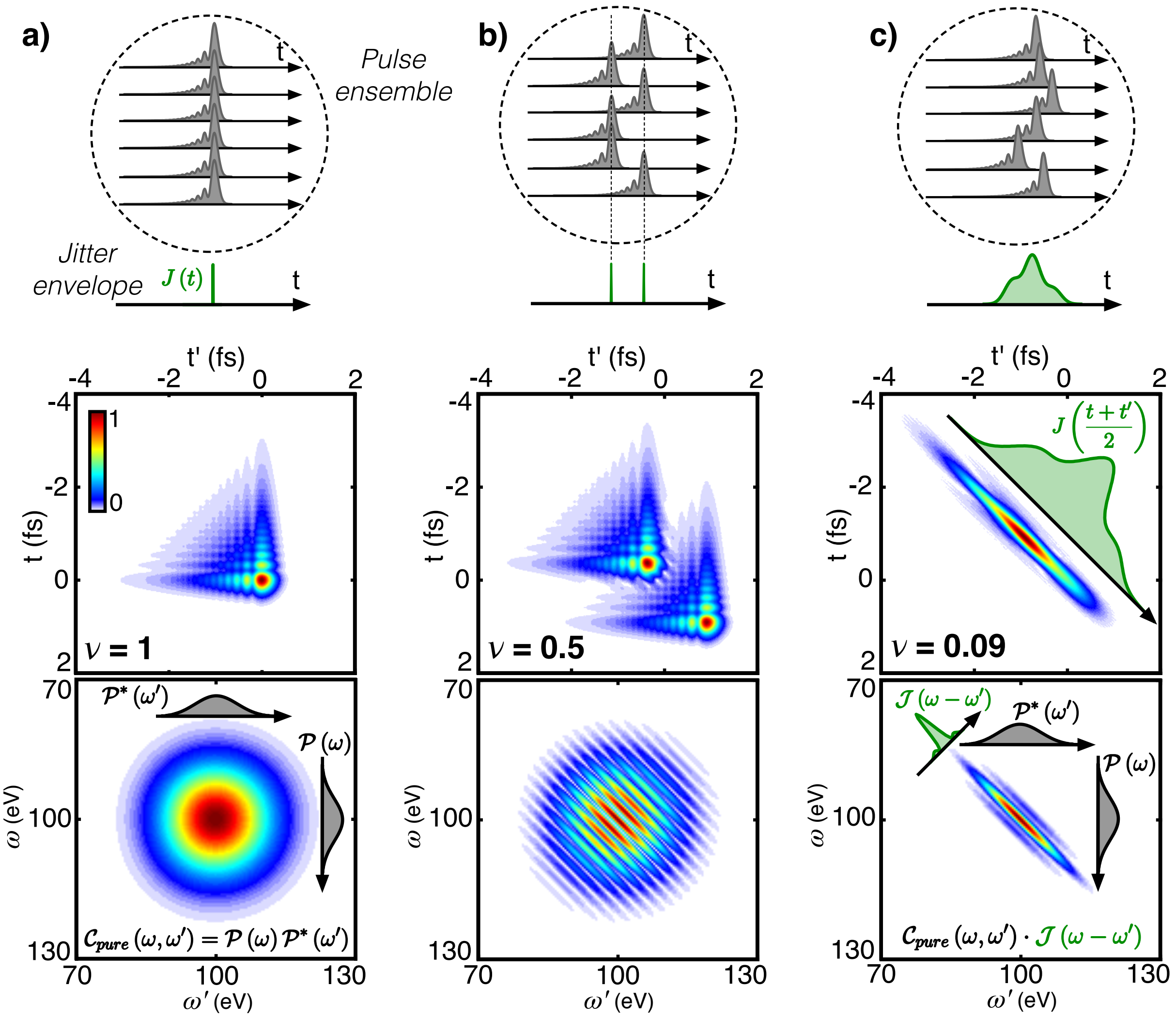

Supplementary Figure 5: **Impact of temporal jitter on the correlation function** The jitter envelope is a) a Dirac function (jitter-free case), b) a double Dirac function, or c) an arbitrary function. In the three scenarios, the pulse ensemble is depicted (upper panel) along with $|C(t,t')|$ (central panel) and $|\mathcal{C}(\omega,\omega')|$ (lower panel).

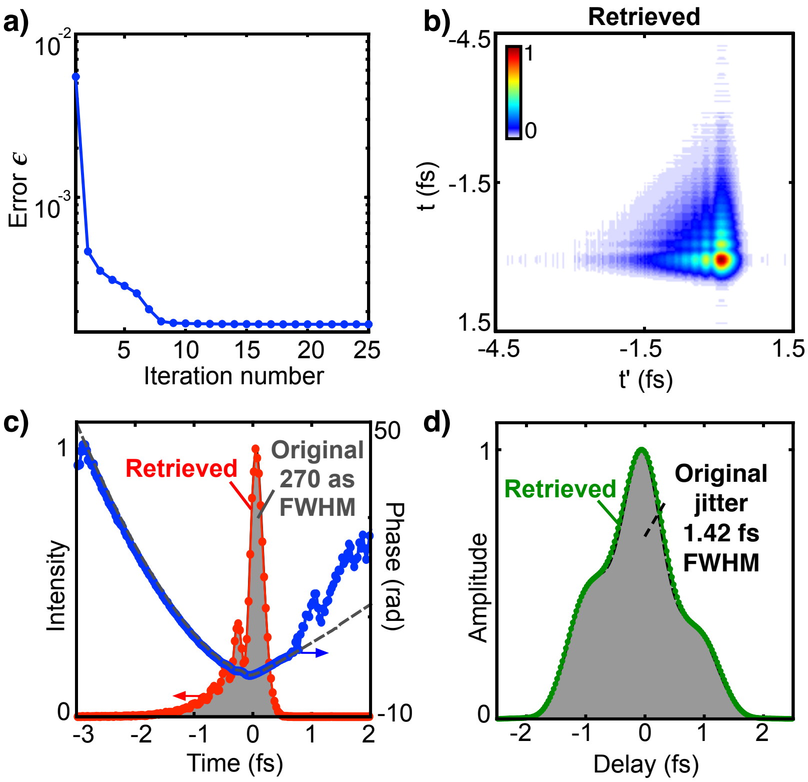

Supplementary Figure 6: **Performances of the blind deconvolution algorithm** a) Evolution of the least squares error ϵ defined in eq. (13) with the number of iterations when the correlation function shown in Supplementary Fig. 5 c) is processed. b) Modulus of the reconstructed jitter-free correlation function $P(t)\,P^*(t')$. c) Reconstructed XUV pulse $P(t)$ and d) jitter envelope $J(t)$.

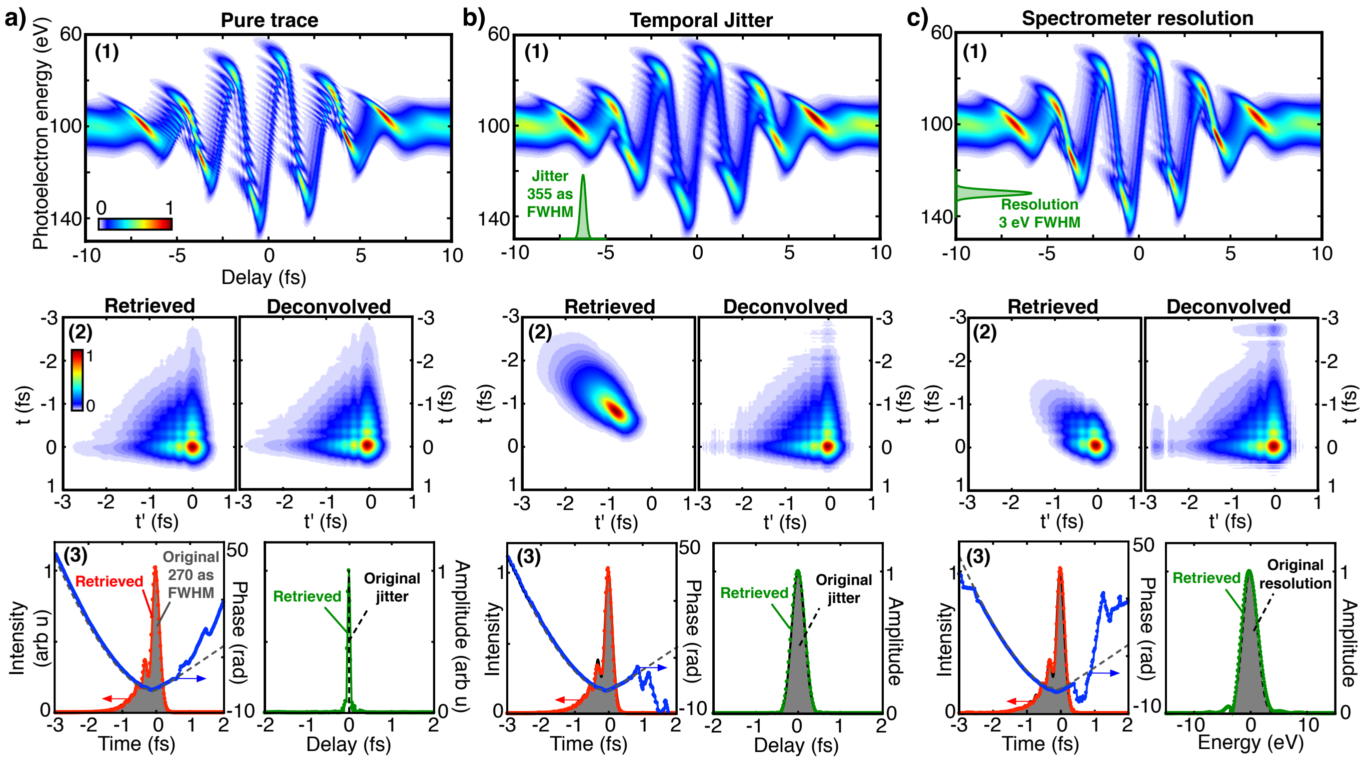

Supplementary Figure 7: **Illustration of the "MSGPA + deconvolution" procedure** Example a) with a pure XUV pulse, b) in the presence of time jitter between the IR and XUV pulses, and c) if accounting for the spectrometer resolution. For the three scenarios, the spectrograms are shown (1) along with the modulus of the retrieved correlation function $|C(t,t')|$ before (left panel) and after (right panel) deconvolution of the source of decoherence (2). In (3) are reported the original (grey curves) and retrieved (red and blue circles) XUV pulse (left panel) as well as the original (dashed line) and retrieved (green circles) jitter and resolution envelope (right panel).

Supplementary Note 1: Mixture retrieval algorithm

General principle. The Principal Components Generalised Projections Algorithm (PCGPA) is a widely used spectrogram inversion algorithm [1]. It iteratively synthesises and then reverses a complex spectrogram $s(\omega,\tau)$, each time fitting it to a given model in the least squares sense. The algorithm stops after a given number of iterations or once the error between the original and retrieved spectrograms becomes sufficiently low. It thus extracts the pulse $P(t)$ and the gate $G(t)$ from a measured spectrogram $S_{meas}(\omega,\tau)$, with

$$S_{meas}(\omega,\tau) = |s(\omega,\tau)|^2 = \left|\underset{t\to\omega}{\mathcal{F}}\left[P(t)\cdot G(t-\tau)\right]\right|^2 \quad (1)$$

and where $\underset{t\to\omega}{\mathcal{F}}$ (resp. $\underset{\omega\to t}{\mathcal{F}^{-1}}$) denotes the Fourier Transform (resp. the inverse Fourier Transform). In order to choose the two constraints applied to the synthesised trace in the real and the Fourier domain, one assumes that the measurement is properly described by eq. (1). Consequently, the constraints are:

- in the spectral domain, that the intensity $|s(\omega,\tau)|^2$ of the synthesised trace matches the measured intensity $S_{meas}(\omega,\tau)$,
- in the time domain, that the matrix $O(t,t-\tau) = \underset{\omega\to t}{\mathcal{F}^{-1}}[s(\omega,\tau)]$ can be summarised by a product of two functions $P(t)\cdot G(t-\tau)$.

Therefore, in the Fourier domain, the solution simply consists in replacing the amplitude of $s(\omega,\tau)$ with the measured one. In the real domain, one rotates the rows of the matrix $O(t,t-\tau)$ to obtain a matrix $O(t,t')$ having an outer product form. If $O(t,t')$ is actually an outer product, then its rank equals one and O has only one non-zero singular value. In the general case, $O(t,t')$ is not an outer product, the solution is therefore to calculate its singular value decomposition and to approximate O as the outer product of the left and right singular vectors associated to the largest singular value, the principal components of O [1]. This is known to be the best outer product fit in the least squares sense. Alternative and less-time consuming techniques, such as the power method [2] or the minimisation of the least-squares error [3, 4], also exist to estimate the principal components.

In the presence of a pulse mixture, the spectrogram corresponds to the incoherent superposition of several pure spectrograms:

$$S_{meas}(\omega,\tau) = \sum_k |s_k(\omega,\tau)|^2 = \sum_k \left|\underset{t\to\omega}{\mathcal{F}}\left[P_k(t)\cdot G(t-\tau)\right]\right|^2 \quad (2)$$

The PCGPA is adapted to partial coherence by parallelising the reconstruction of several pure spectrograms s_k, see Supplementary Fig. 1. However, to ensure that these elementary spectrograms are related to each other, the constraints applied to each s_k must depend on all the other spectrograms. The constraints in the real and Fourier domain are updated following those proposed in Ref. [5] to adapt the difference map algorithm used in ptychography to partial coherence. The new constraints are now:

- in the spectral domain, that the intensity $\sum_k |s_k|^2$ of the synthesised partially coherent trace equals S_{meas},
- in the time domain, that each function $O_k(t,t-\tau)$ represents a product $P_k(t)\cdot G(t-\tau)$.

In this new algorithm, called the Generalised Projections Algorithm for Mixed States (MSGPA), each spectrogram s_k is weighted by an amplitude function $\sqrt{S_{meas}/\sum_k |s_k|^2}$ in the spectral domain. As for the time domain constraint, we first rotate the rows of each matrix $O_k(t,t-\tau)$ to obtain a series of outer product forms. We then concatenate the matrices $O_k(t,t')$, so that the principal components of the resulting matrix correspond to $G(t')$ and to a vector where the pulses $P_k(t)$ are stitched. This ensures that the new guess for the gate is synthesised from the information contained in every O_k.

When the MSGPA stops, one obtains a series of pulses $P_k(t)$ along with the gate. Each pulse $P_k(t)$ being meaningless if treated independently from the rest of the mixture, the retrieved pulses are then converted into statistical quantities, such as the two-time correlation function $C(t,t')$ or the Wigner distribution $W_P(t,\omega)$:

$$C(t,t') = \langle P(t)\,P^*(t')\rangle = \frac{1}{N}\sum_{k=1}^{N} P_k(t)\,P_k^*(t') \quad (3)$$

$$W_P\left(t,\omega\right)=\int dt'C\left(t+\frac{t'}{2},t-\frac{t'}{2}\right)e^{i\omega t'} \tag{4}$$

Initial guesses. For the specific case of FROG-CRAB, the gate is meant to be a pure phase function. The initial guess chosen for the gate is a field having a constant amplitude equal to one and a zero phase. As for the pulse mixture, pulses having various hermite-gauss time profiles are used in order to explore a large variety of pulses.

Convergence. One is able to retrieve partial coherence by giving the MSGPA new degrees of freedom, namely the construction of more than one pure spectrogram. Therefore, the algorithm is less constrained than the PCGPA, and the convergence is observed to require more iterations, typically a few thousands instead of a few hundreds. Moreover, the MSGPA performing the construction of several spectrograms in parallel, each iteration is more time-consuming.

Potential improvements. The various strategies described in Ref. [6] to speed up FROG algorithms, such as the "short-cut generalised projections" or the "over-compensation" technique, should be applicable to mixture retrieval algorithms. Moreover, the retrieval of states mixtures by the construction of several spectrograms in parallel is somewhat independent of the way the spectrogram is generated. Therefore, even though we only treated the case of the PCGP algorithm here, other FROG algorithms can be straightforwardly adapted to state mixtures. As an example, one could imagine using algorithms developed to deal with non-instantaneously nonlinearities [7] to recover mixtures of attosecond pulses with durations approaching the response time of atoms to photoionisation.

Supplementary Note 2: Decoherence deconvolution

Some sources of decoherence have an impact on the correlation function that can be easily modeled. We here analyse such an impact for two common sources of decoherence: the presence of a temporal jitter between the IR and XUV pulses, and the spectral response of the time-of-flight spectrometer. We then present a compact algorithm to separate the source of decoherence from the XUV pulse. Finally, we give numerical examples to illustrate the extraction of the pulse and of the decoherence from a spectrogram.

Impact on the measurement. Consider the presence of time jitter between a pure XUV pulse and the gate. If the ergodicity condition is fulfilled, i.e. if the acquisition time is sufficiently long so that the temporal mean matches the statistical mean, then the measured spectrogram $\langle S(\omega,\tau)\rangle$ reads

$$\langle S(\omega,\tau)\rangle = \int d\delta\tau J(\delta\tau)\cdot S_{pure}(\omega,\tau-\delta\tau) = S_{pure}(\omega,\tau)\otimes J(\tau). \tag{5}$$

S_{pure} represents the jitter-free spectrogram, and $J(\delta\tau)$ corresponds to the probability for the IR/XUV delay τ to deviate from the expected value by an amount $\delta\tau$. Therefore, the impact of time jitter on the spectrogram simply takes the form of a convolution between the pure spectrogram and the jitter envelope $J(\delta\tau)$. The jitter also has a specific impact on the Wigner function $W_P(t,\omega)$ and the correlation function $C(t,t')$ of the XUV pulse mixture. One reformulates eq. (5) to introduce the Wigner distributions,

$$\langle S\rangle = \int d\delta\tau J(\delta\tau)\cdot\left[\iint dt\frac{d\omega'}{2\pi}W_G(t,\omega')\,W_P^{pure}(t-\tau-\delta\tau,\omega-\omega')\right] = \iint dt\frac{d\omega'}{2\pi}W_G(t,\omega')\cdot W_P(t-\tau,\omega-\omega') \tag{6}$$

where $W_P(t,\omega)$ equals $W_P^{pure}(t,\omega)\otimes J(t)$, with $W_P^{pure}(t,\omega)$ the jitter-free Wigner function. Using eq. (4) linking $W_P(t,\omega)$ to $C(t,t')$, one obtains the corresponding two-time correlation function

$$C(t,t') = \int\frac{d\omega}{2\pi}W_P\left(\frac{t+t'}{2},\omega\right)e^{-i\omega(t-t')} = \int d\delta\tau J(\delta\tau)\cdot C_{pure}(t-\delta\tau,t'-\delta\tau) = C_{pure}(t,t')\otimes J\left(\frac{t+t'}{2}\right). \tag{7}$$

In the presence of jitter, $C(t,t')$ thus corresponds to the convolution of the pure correlation function with the jitter envelope. Consequently, in the spectral domain, the correlation function conveniently takes the form of a product between $\mathcal{C}_{pure}(\omega,\omega')$ and $\mathcal{J}(\omega)$, respectively the Fourier Transforms of $C_{pure}(t,t')$ and $J(t)$.

$$\mathcal{C}(\omega,\omega') = \mathcal{C}_{pure}(\omega,\omega')\cdot\mathcal{J}(\omega-\omega') \tag{8}$$

The impact of the spectrometer resolution $\mathcal{R}(\omega)$ on the measurement can be treated equivalently. If the response of the spectrometer is invariant over the spectral range of interest, its effect on the spectrogram can be modeled as a simple convolution of $S_{pure}(\omega,\tau)$ with $\mathcal{R}(\omega)$,

$$\langle S(\omega,\tau)\rangle = S_{pure}(\omega,\tau)\otimes\mathcal{R}(\omega), \tag{9}$$

which leads to the following expressions for the correlation functions in the spectral and time domains,

$$\mathcal{C}(\omega,\omega') = \mathcal{C}_{pure}(\omega,\omega')\otimes\mathcal{R}\left(\frac{\omega+\omega'}{2}\right), \tag{10}$$

$$C(t,t') = C_{pure}(t,t')\cdot R(t-t'), \tag{11}$$

with $R(t)$ the inverse Fourier Transform of $\mathcal{R}(\omega)$.

Ideally, one would like to suppress the influence of jitter or of the spectrometer response in order to recover the XUV pulse hidden in the mixture. Before explaining how this can be done numerically, it is fundamental to understand why the information about the pure pulse is still accessible in the correlation function. Consider the examples in Supplementary Fig. 5. In the three depicted scenarios, the pulse ensemble is composed of identical waveforms suffering from different types of temporal jitter. The chosen pulse has a gaussian spectrum (8 eV RMS) and a spectral phase exhibiting second and third order terms, corresponding to a 270 as pulse FWHM. The associated wave packet is centered at 100 eV.

In the jitter-free scenario shown in Supplementary Fig. 5 a), the two-time (resp. two-frequency) correlation function simply reads as $P(t)\,P^*(t')$ (resp. $\mathcal{P}(\omega)\,\mathcal{P}^*(\omega')$) and exhibits the characteristic symmetries of rank 1 matrices.

In the second case, the arrival time of the pulses in the ensemble can take two possible values, so that the jitter envelope takes the form two Dirac functions, see Supplementary Fig. 5 b). Although unrealistic, this scenario allows one to decompose the impact of jitter on the correlation function. In the time domain, $C(t,t')$ corresponds to the superposition of identical rank 1 patterns $C_{pure}(t,t') = P(t)\,P^*(t')$ translated along the diagonal axis $\frac{t+t'}{2}$ with a probability set by $J\left(\frac{t+t'}{2}\right)$, hence the convolution $C_{pure}(t,t') \otimes J\left(\frac{t+t'}{2}\right)$. In the spectral domain, the convolution is changed into a product, and $\mathcal{C}(\omega,\omega')$ is modulated by the oscillatory function $\mathcal{J}(\omega-\omega')$. Yet, the jitter-free correlation function can easily be guessed, as highlighted by the gaussian function visible in the background.

In the last scenario depicted in Supplementary Fig. 5 c), a jitter envelope with a complex shape (1.4 fs FWHM) is accounted for. Despite the very low purity ($\nu = 0.09$), one can still separate by eye the underlying pure correlation function (grey shaded curves) from the jitter (green shaded curve) in the spectral domain, since $\mathcal{C}(\omega,\omega')$ must follow the form $\mathcal{P}(\omega)\cdot\mathcal{P}^*(\omega')\cdot\mathcal{J}(\omega-\omega')$.

Therefore, the general problem of separating jitter from the XUV pulse in a mixture in fact consists in fitting the two-frequency correlation function to this product form.

Algorithm for the blind deconvolution of the correlation function. One rewrites eq. (8) and affects the indexes i and j respectively to ω and ω' to make apparent the number of measurement points:

$$\mathcal{C}\left(\omega_i,\omega'_j\right) = \mathcal{P}(\omega_i)\cdot\mathcal{P}^*\left(\omega'_j\right)\cdot\mathcal{J}\left(\omega_i-\omega'_j\right) \tag{12}$$

If time jitter is the only source of decoherence, then the correlation function contains a large amount of redundant information. It therefore becomes possible to extract two vectors, the pure XUV pulse $\mathcal{P}_i$ and the jitter envelope $\mathcal{J}_i$, from the matrix $\mathcal{C}_{i,j}$. In fact, we found that it is numerically easier to consider $\mathcal{P}(\omega_i)$ and $\mathcal{P}\left(\omega'_j\right)$ as two distinct functions $\mathcal{P}_i^{(1)}$ and $\mathcal{P}_j^{(2)}$. Therefore, one must split the redundant product between three functions varying along the vertical (i), horizontal (j) and diagonal axes $(i-j)$. This situation is essentially the same as the one faced in the PCGP and MSGP algorithms when applying the product constraint to generate two estimates for $P(t)$ and $G(t)$, as shown in Supplementary Fig. 1. One can thus adopt a similar solution such as the least-squares minimisation. One wants to identify the three functions $\mathcal{P}_i^{(1)}$, $\mathcal{P}_i^{(2)}$ and $\mathcal{J}_i$ which minimise the least squares error ϵ defined as

$$\epsilon = \sum_{i,j}\left|\mathcal{C}_{i,j} - \mathcal{P}_i^{(1)}\mathcal{P}_j^{*(2)}\mathcal{J}_{i-j}\right|^2. \tag{13}$$

One differentiates ϵ with respect to these three functions and sets these derivatives to zero. This leads to a set of three coupled equations:

$$\mathcal{P}_i^{(1)} = \frac{\sum_j \mathcal{C}_{i,j}\mathcal{P}_j^{(2)}\mathcal{J}_{i-j}^*}{\sum_j\left|\mathcal{P}_j^{(2)}\mathcal{J}_{i-j}\right|^2} \tag{14}$$

$$\mathcal{P}_j^{*(2)} = \frac{\sum_i \mathcal{C}_{i,j}\mathcal{P}_i^{(1)*}\mathcal{J}_{i-j}^*}{\sum_i\left|\mathcal{P}_i^{(1)}\mathcal{J}_{i-j}\right|^2} \tag{15}$$

$$\mathcal{J}_j = \frac{\sum_i \mathcal{C}_{i,i+j}\mathcal{P}_i^{(1)*}\mathcal{P}_{i+j}^{(2)}}{\sum_i\left|\mathcal{P}_i^{(1)}\mathcal{P}_{i+j}^{(2)}\right|^2} \tag{16}$$

One proceeds iteratively to solve this system. Starting from random guesses for $\mathcal{P}_j^{(2)}$ and $\mathcal{J}_j$, one deduces from eq. (14) an estimate of $\mathcal{P}_i^{(1)}$. The latter estimate is then inserted into eq. (15) along with the initial guess chosen for $\mathcal{J}_j$, leading to an estimate for $\mathcal{P}_j^{(2)}$. The field associated to $\mathcal{J}_j$ is finally updated by applying eq. (16). By repeating this process several times, one progressively refines the estimates of the three fields. This algorithm has been tested

with experimental data in the context of interferometric measurements, and has proven to be robust against noise and to converge in very few iterations [8].

We now process the two-frequency correlation function $\mathcal{C}(\omega, \omega')$ shown in Supplementary Fig. 5 c) with this algorithm. As shown in Supplementary Fig. 6 a), the algorithm converges in less than 10 iterations of eqs. (14,15,16), which takes a few seconds in practice. Increasing further the number of iterations does not decrease ϵ, the convergence being limited by the noise background originally added to the correlation function.

However, the three fields retrieved with this algorithm are known to suffer from one ambiguity [8]: $\mathcal{P}^{(1)}(\omega) \cdot \mathcal{P}^{(2)*}(\omega') \cdot \mathcal{J}(\omega - \omega') = \mathcal{P}^{(1)}(\omega)\, e^{\gamma\omega} \cdot \mathcal{P}^{(2)*}(\omega')\, e^{-\gamma\omega'} \cdot \mathcal{J}(\omega - \omega')\, e^{-\gamma(\omega-\omega')}$, where $\gamma = \gamma' + i\gamma''$ is a complex number. Each function is thus properly retrieved up to linear phase term $e^{i\gamma''\omega}$ and up to an exponential term $e^{\gamma'\omega}$ affecting the field modulus. The linear phase term is unimportant since it corresponds to an absolute shift in the time domain, which is a meaningless quantity. However, the term $e^{\gamma'\omega}$ needs to be removed. To do so, we take advantage of the fact that $\mathcal{P}^{(1)}(\omega)$ and $\mathcal{P}^{(2)}(\omega)$ must correspond to the same function $\mathcal{P}(\omega)$. In fact, if $\mathcal{P}(\omega)$ is obtained by estimating the geometric mean of the two pulses, $\mathcal{P}(\omega) = \sqrt{\mathcal{P}^{(1)}(\omega) \cdot \mathcal{P}^{(2)}(\omega)}$, then the terms $e^{\gamma'\omega}$ naturally cancel out, providing an unambiguous estimate for $\mathcal{P}(\omega)$. Equivalently, calculating the ratio $\sqrt{\mathcal{P}^{(1)}(\omega)/\mathcal{P}^{(2)}(\omega)} = e^{\gamma'\omega}$ allows one to isolate the exponential term which can then be used to obtain a corrected estimate for $\mathcal{J}(\omega)$. As a last step, a simple Fourier Transform of $\mathcal{P}(\omega)$ and $\mathcal{J}(\omega)$ allows one to obtain the time profile of the XUV pulse $P(t)$ as well as the jitter envelope $J(t)$. The fields retrieved with this procedure along with the corresponding jitter-free correlation function $P(t)\, P^*(t')$ are shown in Supplementary Fig. 6 (b-d), and agree very well with the original fields.

A similar procedure can be applied to separate the influence of the spectrometer resolution from the XUV pulse. In this scenario, the correlation function adopts a product form $P^{(1)}(t_i) \cdot P^{(2)*}(t'_j) \cdot R(t_i - t'_j)$ in the time domain, see eq. (11). One must therefore replace $\mathcal{C}(\omega_i, \omega'_j)$ with $C(t_i, t'_j)$ in eqs. (14,15,16) in order to retrieve both the XUV field $P(t_i)$ and $R(t_i)$, the Fourier Transform of $\mathcal{R}(\omega)$.

Numerical examples. We now illustrate the full "decoherence removal" procedure starting the processing from the spectrogram, see the three examples depicted in Supplementary Fig. 7. A pure spectrogram is generated and then convolved with a given filter along the delay or spectral axis to simulate the presence of time jitter or the response of the spectrometer (1). The spectrogram is then processed with the MSGPA to retrieve the correlation function $C(t, t')$ associated to the mixture of XUV pulses. Finally, the correlation function is processed with the help of the blind deconvolution algorithm (2) in order to retrieve the underlying XUV pulse (3).

The XUV pulse is the same 270 as pulse as in Supplementary Figs. 5 and 6. The laser pulse is a 800 nm Fourier-limited gaussian pulse (5 fs intensity FWHM) with an intensity of 8 TW cm^{-2}. In each case, noise is added to the final spectrogram with a level equal to 1% of the peak intensity of the photoelectron spectrum in the absence of laser field. The spectrograms, having a size of 512×512 points, are processed with the MSGPA using 20 modes, i.e. 20 pulses $P_k(t)$, to describe the mixture. The algorithm is stopped after 10^4 iterations. The deconvolution is then performed after 5 iterations of eqs. (14,15,16).

In the first scenario, no source of decoherence is added, see Supplementary Fig. 7 a). The pure spectrogram contains fine fringes due to the third-order term in the spectral phase of the attosecond pulse. Consequently, the retrieved correlation function $P(t)\, P^*(t')$ corresponds to a matrix of rank 1, and the jitter deconvolution algorithm has no effect on it. The amplitude and phase of the pulse are well retrieved, along with the jitter envelope which corresponds to a Dirac distribution.

In the second case depicted in Supplementary Fig. 7 b), a temporal jitter with a gaussian envelope of 355 as FWHM is added to the pure spectrogram in a). As a consequence, the fringe structure is severely blurred and the retrieved correlation function is smoothed by the jitter. The pulse and jitter envelope recovered by the deconvolution algorithm nicely match the original ones.

In order to account for the resolution of the time-of-flight electron spectrometer, the pure spectrogram is convolved along the spectral axis with a gaussian function (3 eV FWHM), resulting in the disappearance of the fringes in the spectrogram, see Supplementary Fig. 7 c). In such a scenario, $C(t, t')$ reads $P(t)\, P^*(t')\, R(t - t')$, see eq. (11). This product form is equivalent to the one described in the lower panel in Supplementary Fig. 5 c) in the case of jitter. Consequently, the retrieved correlation function exhibits the same structure as C_{pure} near the diagonal axis, as highlighted by the characteristic bounces. This structure rapidly disappears as one moves away from the diagonal due to the product with the gaussian function $R(t - t')$. The deconvolution algorithm is then applied. Despite a small artifact in the wings of C_{pure} where the relevant signal can no longer be distinguished from the noise, the algorithm efficiently separates the influence of the pulse and of the response of the spectrometer, as shown in Supplementary Fig. 7 c) (3).

Limitations of the approach. As illustrated in Supplementary Fig. 6, the deconvolution algorithm works very well even with mixtures having a very low purity, i.e. even with very large jitters. However, in that case, the challenge is to make sure that the correlation function can be accurately reconstructed from the spectrogram. As explained in the main text, the lower the purity of the mixture to retrieve, the larger the required laser intensity. In the examples given here, an intensity of $8\,\mathrm{TW\,cm^{-2}}$ was required to recover a 270 as pulse with a jitter of 355 as. The same reconstruction with a jitter of 1.4 fs, as used in Supplementary Fig. 6, would probably require a laser intensity of a few tens of $\mathrm{TW\,cm^{-2}}$. In practice, parasitic phenomena, such as multi-IR-photon ionisation, would compete with the two color XUV-IR ionisation, potentially preventing the reconstruction.

Generalisation to other sources of decoherence. The procedure described here can be extended to other sources of decoherence than jitter and the spectrometer response. The general approach to follow can be decomposed in three main steps:

1. Identify the potential sources of decoherence and the mathematical form of their impact on $C\left(t,t'\right)$,
2. Establish a numerical model $C_{i,j}^{model}\left(\alpha_1,...,\alpha_N\right)$ for the partially coherent correlation function, where $\left(\alpha_1,...,\alpha_N\right)$ represent a small number of parameters summarising the mixture, e.g. the two vectors $\mathcal{P}_i$ and $\mathcal{J}_i$ in the case of jitter, bearing in mind that the fitting procedure works so long as the system remains overdetermined.
3. Fit the measured correlation function to this model using an optimisation algorithm with a merit function such as $\sum_{i,j}\left|C_{i,j}^{measured}-C_{i,j}^{model}\left(\alpha_1,...,\alpha_N\right)\right|^2$ in order to recover the parameters $\left(\alpha_1,...,\alpha_N\right)$.

Supplementary Methods

Two-color photoionisation. The two-color XUV-IR photoionisation can be efficiently described under the Strong-Field Approximation (SFA). In the frame of the first order perturbation theory with the single active electron approximation, the SFA gives the following expression in atomic units for the amplitude $a_{\mathbf{p}}$ of the transition from the ground state to the state $|\mathbf{p}\rangle$ of momentum $\mathbf{p}$ in the continuum [9]:

$$a_{\mathbf{p}}(\tau) = -i \int_{-\infty}^{+\infty} dt \mathbf{d}_{\mathbf{p}'(t)} \mathbf{E}_{XUV}(t-\tau) \cdot e^{i \int_t^{+\infty} [I_p - \mathbf{p}'^2(t')/2] dt'} \tag{17}$$

In this equation, τ stands for the delay between the IR and XUV fields, I_p is the ionisation potential of the atomic species, $\mathbf{p}'(t) = \mathbf{p} + \mathbf{A}(t)$ represents the instantaneous momentum, $\mathbf{A}(t)$ is the vector-potential of the laser field $\mathbf{E}_{IR}(t) = -\partial \mathbf{A}/\partial t$, $\mathbf{E}_{XUV}(t)$ corresponds to the XUV electric field, and $\mathbf{d}_{\mathbf{p}}$ the dipole matrix element of the transition from the ground state to the state $|\mathbf{p}\rangle$. The accessible quantity in practice is the square modulus of $a_{\mathbf{p}}$, that is the energy spectrum of the photoelectrons. Introducing the kinetic energy of the photoelectrons $W = \mathbf{p}^2/2$, one rewrites eq. (17) to make the spectrogram equation appear:

$$\langle S(W,\tau)\rangle = \left\langle \left| -i \int_{-\infty}^{+\infty} dt \mathbf{d}_{\mathbf{p}'} \mathbf{E_{XUV}}(t-\tau) \cdot e^{i\phi(\mathbf{p},t)} \cdot e^{i(W+I_p)t} \right|^2 \right\rangle = \left\langle \left| \mathcal{F}_{t \to W} \left[P(t-\tau) \cdot G(t) \right] \right|^2 \right\rangle \tag{18}$$

As in the main text, $\langle\rangle$ denotes the averaging of the spectrogram over an ensemble. The pulse $P(t)$ corresponds to the electron wave packet $\mathbf{d}_{\mathbf{p}'} \mathbf{E_{XUV}}(t)$, and the gate $G(t)$ equals $\exp(i\phi(\mathbf{p},t))$, where $\phi(\mathbf{p},t) = -\int_t^{+\infty} dt' \mathbf{p} \cdot \mathbf{A}(t') + A(t')^2/2$. Note that we do not apply the "central momentum approximation" here: $\phi(\mathbf{p},t)$ is evaluated for each momentum $\mathbf{p}$, and the final amplitude $a_{\mathbf{p}}$ is obtained by integrating numerically the function $P(t-\tau) \cdot e^{i\phi(\mathbf{p},t)} \cdot e^{i(W+I_p)t}$ over t. This procedure is used to calculate all the photoelectron spectra presented in the article. Moreover, the dipole matrix element $\mathbf{d}_{\mathbf{p}'}$ has been assumed to be constant (flat cross-section and constant or negligible atomic phase) across the XUV pulse bandwidth. This is equivalent to saying that the electron wave packet is an exact replica of the XUV pulse, up to an energy shift I_p. If the dipole matrix element is known beforehand, this procedure could be improved by dividing the retrieved wave packet in the frequency domain $d_{p'} E_{XUV}(W)$ with $d_{p'}$ in order to recover the optical wave packet $E_{XUV}(\omega + I_p)$ [10]. Finally, we consider that the electrons are collected along the direction of polarisation of the laser field, which enables one to replace $\mathbf{p} \cdot \mathbf{A}$ with pA in the gate expression.

Simulation of the RABBIT measurement in Fig. 2. The mixture used to simulate the RABBIT measurement was formed by the incoherent averaging of the attosecond pulse trains $P_k(t)$ in Supplementary Fig. 2 a), i.e. $C(t,t') = \langle P_k(t) P_k^*(t')\rangle_k$. All the pulses in the mixture have a common spectrum composed of gaussian harmonics (0.6 eV FWHM, 1.55 eV fundamental frequency) contained in a 9 eV FWHM gaussian envelope centered on harmonic 31. In order to illustrate the ability of Mixed-FROG to retrieve highly mixed states, we used exaggerated variations of the group delay dispersion (GDD) and of the absolute delay of the pulse trains. The GDD (resp. the absolute group delay) of the pulse trains varies linearly with k from 10^5 to $2 \cdot 10^5\,\mathrm{as}^2/\mathrm{rad}$ (resp. from -1 to $+1$ fs). This results in a mixture with a very low purity ν of 0.26.

The photoelectron spectra were then simulated using argon as detection gas and a 8 fs (intensity FWHM) 800 nm gaussian dressing pulse. The traces consisted of 256 × 256 traces. In the case where the laser intensity equals $0.02\,\mathrm{TW\,cm}^{-2}$, the spectrum and group delay retrieved with RABBIT and with FROG-CRAB after 10^4 iterations of the PCGPA appear in Supplementary Fig. 2 b). In order to identify the optimal mode number for the mixed state reconstruction, we processed the same RABBIT trace using various numbers of modes, see Supplementary Fig. 3 a). Following the definition of the R-factor used in ptychography to monitor the convergence of the difference map algorithm [11], we define the error R between the original $\langle S\rangle_O$ and retrieved $\langle S\rangle_R$ spectrograms as

$$\mathrm{R} = \frac{\Sigma_{(\omega,\tau)} \left| \sqrt{\langle S\rangle_O} - \sqrt{\langle S\rangle_R} \right|}{\Sigma_{(\omega,\tau)} \sqrt{\langle S\rangle_O}}. \tag{19}$$

When using a single mode, which corresponds to the FROG-CRAB reconstruction in Supplementary Fig. 2 b), the error rapidly stagnates, highlighting that partial coherence is not properly accounted for. Using two modes dramatically reduces the error, which confirms the presence of a pulse mixture. Convergence can be slightly improved with 5 modes. However, using 10, 20 or 30 modes makes hardly any difference. The same conclusion can be drawn from the evolution of the retrieved temporal marginals shown in Supplementary Fig. 3 b). $\left\langle |P(t)|^2 \right\rangle$ no longer changes

when more than 10 modes are used. Using 10 modes thus constitutes the best tradeoff between computation time and quality of the reconstruction of the mixture. This also shows that increasing the number of modes does not enable one to properly recover the original mixture shown in Fig. 2 in the main text. This confirms that the complete information about the mixture is fundamentally not present in the spectrogram when the RABBIT trace is obtained with a low intensity dressing field. The missing information can only be obtained by imprinting a stronger modulation onto the electron wave packet, that is by increasing the laser intensity. Therefore, the traces obtained for various laser intensities in Fig. 2 d) in the main text were processed with the MSGPA, using 10 modes and 10^4 iterations, and starting from the same initial state to ensure a relevant comparison.

Simulation of the attosecond pulse in Fig. 3. The photoelectron spectra were simulated using a replica of the driving pulse as the dressing beam (intensity $5\,\mathrm{TW\,cm^{-2}}$) and argon as detection gas. The spectrograms were first calculated at each radius r and then averaged to account for the spatial integration $\langle S\left(W,\tau\right)\rangle = 2\pi\int S\left(W,\tau,r\right)rdr$. The resulting 512×512 spectrogram was then processed using 10 modes and 10^4 iterations of the MSGPA. The computation time was about 2 hours on a standard computer. The convergence is depicted in the supplementary movie.

Simulation of the Free-Electron Laser pulse in Fig. 4. The two-color photoionisation process was simulated using the ionisation potential of argon. The IR pulse has a gaussian spectrum centered at 800 nm (19 fs Fourier-limit) and a third order term ($5\cdot10^4\,\mathrm{fs^3/rad^2}$) was added on the spectral phase, resulting in a duration of 20 fs FWHM. The pulse intensity in the gas jet equals $2\,\mathrm{TW\,cm^{-2}}$. The final spectrogram consisted in a 1024×1024 trace. In order to simulate the absence of CEP stabilisation of the dressing laser, the trace was first averaged over 20 values of the carrier-envelope-phase of the IR pulse equally distributed between 0 and 2π, resulting in the trace shown in the left panel of Fig. 4 a) in the main text. Using eq. (5), the trace was then convolved with a 20 fs FWHM jitter gaussian envelope to account for the laser/XUV jitter, leading to the trace in the right panel of Fig. 4 a) in the main text. The reconstruction was performed over 30 modes and $5\cdot10^4$ iterations of the MSGPA. 25 iterations of eqs. (14,15,16) were then necessary to deconvolve the obtained correlation function and retrieve the FEL pulse and jitter envelope.

We depict in Supplementary Fig. 4 the evolution of the error R defined in eq. (19) with respect to the iteration number in the case of the Mixed-FROG reconstruction (MSGPA with 30 modes) and of the FROG-CRAB reconstruction (monomode PCGPA) depicted in Fig. 4 (d-e) in the main text. The same observation as in the case of the RABBIT reconstruction in Supplementary Fig. 3 a) can be made. While R rapidly stagnates at a value of ~ 0.15 in the single mode case, it continuously decreases down to $\sim 5\cdot10^{-3}$ after $5\cdot10^4$ iterations when 30 modes are used. This highlights that in the presence of jitter the spectrogram is much more accurately reproduced if partial coherence is accounted for.

Supplementary References

[1] Kane, D. J., Rodriguez, G., Taylor, A. J. & Clement, T. S. Simultaneous measurement of two ultrashort laser pulses from a single spectrogram in a single shot. *J. Opt. Soc. Am. B*, **14,** 935-943 (1997).
[2] Kane, D. J. Real-time measurement of ultrashort laser pulses using principal component generalized projections. *IEEE J. Sel. Top. Quant. Electron.*, **4,** 278-284 (1998).
[3] Trebino, R. *et al.* Measuring ultrashort laser pulses in the time-frequency domain using frequency-resolved optical gating. *Rev. Sci. Instrum.*, **68,** 3277-3295 (1997).
[4] Gagnon, J. & Yakovlev, V. S. The robustness of attosecond streaking measurements. *Opt. Express*, **17,** 17678-17693 (2009).
[5] Thibault, P. & Menzel, A. Reconstructing state mixtures from diffraction measurements. *Nature*, **494,** 68-71 (2013).
[6] Trebino, R. Frequency-Resolved Optical Gating: The Measurement of Ultrashort Laser Pulses. *Springer* (2000).
[7] DeLong, K. W., Ladera, C. L., Trebino, R., Kohler, B. & Wilson, K. R. Ultrashort-pulse measurement using noninstantaneous nonlinearities: Raman effects in frequency-resolved optical gating. *Opt. Lett.*, **20,** 486-488 (1995).
[8] Bourassin-Bouchet, C., Mang, M. M., Gianani, I. & Walmsley, I. A. Mutual interferometric characterization of a pair of independent electric fields. *Opt. Lett.*, **38,** 5299-5302 (2013).
[9] Quéré, F., Mairesse, Y. & Itatani, J. Temporal characterization of attosecond XUV fields. *J. Mod. Opt.*, **52,** 339-360 (2005).
[10] Bhardwaj, S., Keathley, P. D., Moses, J., Laurent, G. & Kärtner, F. An improved attosecond-pulse characterization based on the FROG-CRAB technique. In CLEO: Science and Innovations (pp. JTu4A-2). Optical Society of America. (2014, June).
[11] Thibault, P., Dierolf, M., Bunk, O., Menzel, A. & Pfeiffer, F. Probe retrieval in ptychographic coherent diffractive imaging. *Ultramicroscopy*, **109,** 338-343 (2009).
